# A Case of Recurrent Painful Ophthalmoplegic Neuropathy

**DOI:** 10.3389/fneur.2020.00440

**Published:** 2020-06-04

**Authors:** Yaping Yan, Bo Zhang, Zhuocong Lou, Kaiming Liu, Ming Lou, Meiping Ding, Yongfeng Xu

**Affiliations:** ^1^Department of Neurology, Second Affiliated Hospital, College of Medicine, Zhejiang University, Hangzhou, China; ^2^Department of Surgery, Second Affiliated Hospital, College of Medicine, Zhejiang University, Hangzhou, China; ^3^Department of Neurology, Affiliated Hospital of Shaoxing University, Shaoxing, China

**Keywords:** migraine, repeated oculomotor paralyzes, diffusion weighted imaging (DWI), schwannoma, demyelination

## Abstract

Ophthalmoplegic migraine (OM) is characterized by recurrent episodes of headache with unilateral ophthalmoplegia due to paresis of cranial nerve III, IV, or VI. The recent revision to the International Headache Classification has reclassified it as recurrent painful ophthalmoplegic neuropathy (RPON). However, it is of note that the presentation of oculomotor nerve tumors may mimic RPON. Here, we report the case of a patient presenting with recurrent migraine and oculomotor palsy with several specific magnetic resonance imaging (MRI) findings. The patient was initially diagnosed with migraine 15 years ago, but since 10 years ago, his symptoms had evolved to include repeated oculomotor paralyzes. Before this attack, the patient did eventually recover completely each time after the initial episode. MRI performed during this attack revealed a nodular enhancing lesion described as schwannoma of the left oculomotor nerve, and on diffusion-weighted imaging (DWI), the nerve was isointense to the midbrain. The nodular enhancement became weaker, and the nerve's signal on DWI disappeared 3 months later as the patient's symptoms resolved mostly. This is the first case of RPON demonstrating an obvious change in signal of the affected nerve on DWI during the attack and remission.

## Introduction

Ophthalmoplegic migraine (OM) was first reported by Charcot in 1890, described as migraine with oculomotor nerve palsy (1). More than 100 years later, OM was included as a migraine variant in the first edition of the International Headache Classification (IHCD- I) (2). Then, in 2004, the disorder was reclassified as one of the cranial neuralgias in ICHD-II due to the enhancement and thickening of the involved nerve in the brain as observed on magnetic resonance imaging (MRI) (3). In 2013, OM was replaced by recurrent painful ophthalmoplegic neuropathy (RPON) in IHCD-3 based on headache, subsequent cranial neuropathies, MRI features, and the fact that some cases showed response to corticosteroid treatment (4). However, the etiology remains controversial, but mainly involves schwannoma or recurrent bouts of demyelination of the affected nerve (5, 6).

Reduction or complete resolution of focal enhancement in the cisternal portion of the involved nerve is observed in most RPON patients and in rare patients with schwannoma (7–9). Instead, persistent nodular enhancement of the lesion is seen in most patients with schwannoma (10, 11). Here, we report a case of recurrent migraine and oculomotor palsy in which MRI showed enhancement of the oculomotor nerve with visible DWI signal at the midbrain exit while the patient was symptomatic. Follow-up MRIs more than 4 months after the resolution of the latest episode demonstrated reduced enhancement and resolution of the DWI signal.

## Case Presentation

**Figure 1:**
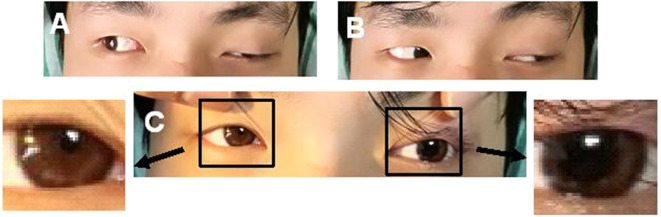
A case of left ptosis, difficulty in seeing inward **(A)**, and left pupil enlargement **(C)**. The case had normal left abduction **(B)**.

**Figure 2:**
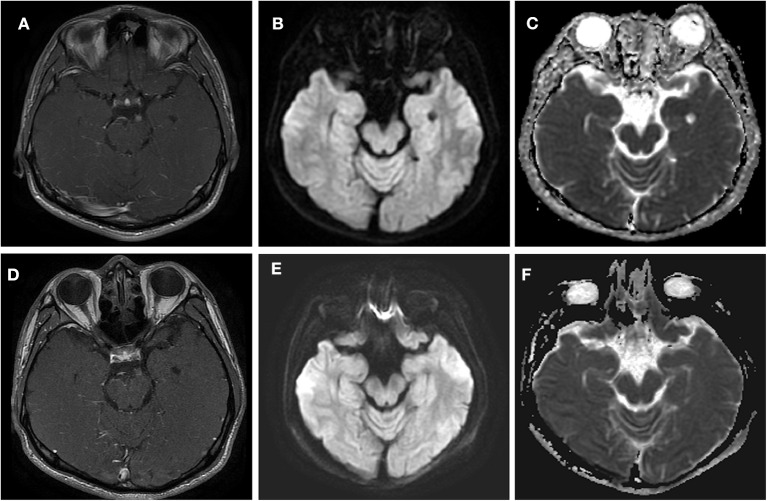
Axial T1 contrast-enhanced MRI (44 days after headache) and DWI (30 days after headache) through the point at which the oculomotor nerve emerges from the midbrain in this patient with left oculomotor ophthalmoplegic migraine. **(A–C)** Acute phase. The MRI shows a nodular, enhancing lesion in the cisternal segment of the left third nerve (arrow) in **(A)**. In **(B,C)**, a signal was demonstrated at the midbrain of the left-sided third cranial nerve on DWI and ADC. **(D–F)** Quiescent phase, taken 7 weeks after the left ptosis and diplopia had resolved. **(D)** Demonstrates the decreased enhancement. **(E,F)** Demonstrate that the signal had disappeared at this point on DWI and ADC.

**Figure 3:**
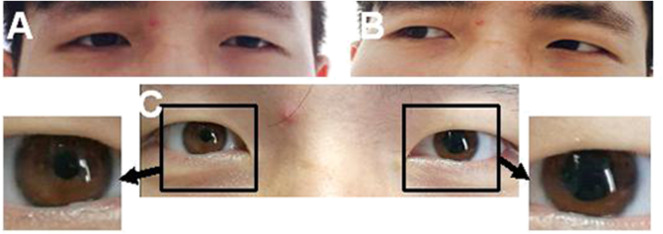
Relatively dilated left pupil **(C)** with recovered extra-ocular movement **(A,B)**.

A 24-year-old man was admitted to our hospital with a complaint of diplopia. Before admission, he had been experiencing migraine and then concurrent oculomotor paralysis for 1 month. He was treated with prednisolone (40 mg/d) for 4 days but without any improvement. He denied a family history of migraine. General physical examination was normal. Neurological examination revealed a complete paralysis of the left third cranial nerve (Figure 1). Serologic and cerebrospinal fluid (CSF) studies were normal. MRI revealed focal enhancement of the cisternal segment of the left oculomotor nerve (Figure 2A). Meanwhile, the affected nerve was isointense to the midbrain on DWI and apparent diffusion coefficient (ADC) maps (Figures 2B,C). He received intravenous methylprednisolone (80 mg/d) for 8 days followed by prednisolone taper for 15 days but still experienced no immediate improvement. Gradually, over the course of the next 3 months, the patient's left-sided ptosis and diplopia showed complete resolution although the left pupil remained relatively dilated and poorly reactive to light (Figure 3). Repeated MRI revealed relatively weaker enhancement with no signal visible on DWI and ADC maps (Figures 2D–F).

**Figure 4:**
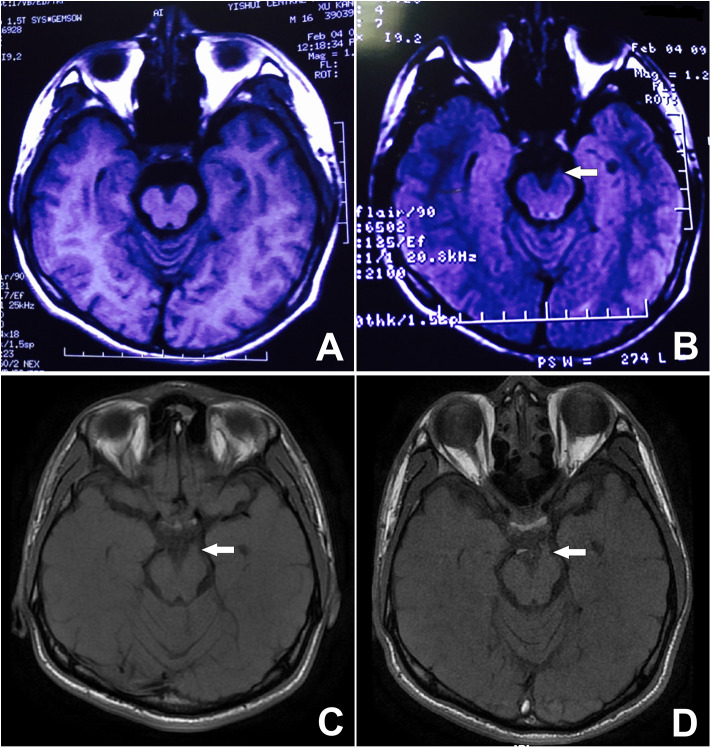
Unenhanced MRI from 9 years prior showing minor thickening of the distal cisternal portion of the left-sided third cranial nerve on T2 FLAIR. See arrow **(B)**. The signal on T1 is obvious, during both the attack and remission. See arrow **(C,D)** although it is not visible on the T1 image from 9 years ago **(A)**.

Back to the patient's history, he had been suffering from monthly episodes of migraine for the past 15 years, lasting for 24 h each time. Then, 10-years ago, he started experiencing diplopia and a left-sided ptosis after resolution of the headache and nausea every 2 months, which persisted for 1 week each time. A review of a previous MRI performed at another hospital 9-years earlier, when the patient was aged 15, showed slight thickening of the left third cranial nerve at the site of exit from the midbrain, especially on T2 FLAIR (Figure 4B). At that time, the patient was able to prevent the development of oculomotor paralyzes by taking aminopyrine-caffeine tablets immediately when the headache occurred. However, the time to resolution of the oculomotor paralysis had begun to increase to usually 2–3 weeks, and the frequency of the attacks was reduced to 3–4 times a year. During the past 15-years, although the symptoms resolved completely each time except for this attack, the lesion had increased in size on T1 since his initial presentation (Figures 4A,C,D).

## Discussion

As Carlow have reported, enhancement of the cisternal segment of the third nerve was usually detected during an attack, resolving several weeks later when the patient's symptoms had also been relieved. Based on this observation, Carlow proposed that the diagnostic criteria for oculomotor OM should include the presence of post-contrast enhancement on MRI and thickening of the third nerve during an attack with less dramatic enhancement observable during remission (12). Here, we report a male adolescent who had been suffering from recurrent painful ophthalmoplegic neuropathy (RPON) for 10-years. Consistently, several other similar cases have been reported recently. The image feature of these patients on MRI with contrast was quite similar to that in the present study (6, 13). Additionally, our case also displayed an obvious signal change in the affected nerve on DWI and ADC maps during the episode compared to that during remission. To our knowledge, this is the first reported case accompanied by such neuroimaging documentation.

With respect to the pathophysiology of this disease entity, the underlying mechanism remains controversial because of lack of autopsy or surgical resection (14). However, some possible theories have been proved histopathologically, mainly including infectious neuritis, ischemia of the third cranial nerve, recurrent demyelination-remyelination, and schwannoma (15–18). As the cerebrospinal fluid and serum test was normal, there was no evidence to support an infectious process in the current case. And the lesion on MRI image did not favor the ischemia mechanism.

The repeated demyelinating theory has gained favor in recent years (6, 16, 18), which is supported by features such as the recurrent episodes; nearly complete remission every time; and transient, reversible MRI contrast enhancement of the oculomotor nerve. Our patient refused biopsy as the symptom could resolve every time. Therefore, histopathological evidence to support this hypothesis was not available.

As for the tumor pathogenesis, it has been reported that transient or recurrent oculomotor nerve deficits may be the primary manifestation in some cases of cranial nerve schwannoma (10). Recently, Petruzzelli et al. have reported a case of a 16-year-old boy with a diagnosis of OM initially but which turned out to have the presentation of a schwannoma after 7-year follow-up (5). In her review, three cases of schwannoma of the affected nerve had been reported, which included a long history of follow-up, and only one was histologically proven (17). It was reported that more than 60% of patients with oculomotor schwannoma treated by surgery developed more severe opthalmoplegia postoperatively (19). So few cases of RPON accept surgery (15–18). However, the increased thickening of the oculomotor nerve observed after 9 years of RPON attacks may serve as evidence in support of the tumor theory. This hypothesis extends the inflammatory theory, proposing that repeated bouts of inflammation may lead to cycles of demyelination and remyelination of the affected nerve, and such a process may encourage the transformation of Schwann cells to schwannoma (5). Yet, as Kim et al., Akimoto et al., and Shin et al., have proposed, follow-up surveillance via MRI is required to exclude tumors, especially in patients with suspected RPON with persistent post-contrast enhancement during times of remission (10, 11, 20). However, the enhancement in this case that we describe is weaker during remission than that during the acute episode, which is similar to what was observed in another reported case in which a nodular enhancing lesion was already visible and of similar size (8). As such, whether the nodular lesion in fact represented a schwannoma remains controversial.

In conclusion, the exact underlying nature of the findings in this patient remains unknown due to the lack of histopathological evidence. Further imaging studies incorporating DWI sequence analysis and serial MRI follow-up of a greater number of cases are required to clarify whether RPON may be a risk factor for the development of schwannoma over an extended period.

## Data Availability Statement

All datasets generated for this study are included in the article/supplementary material.

## Ethics Statement

The studies involving human participants were reviewed and approved by The Second Affiliated Hospital, Zhejiang University School of Medicine. The patients/participants provided their written informed consent to participate in this study. Written informed consent was obtained from the individual(s) for the publication of any potentially identifiable images or data included in this article.

## Author Contributions

YY, ZL, KL, ML, MD, and YX were responsible for diagnosis and clinical evaluation. YY, ML, MD, and YX collected and analyzed MRI data. YY and BZ wrote the manuscript. YY, BZ, and YX contributed to the revision of the manuscript.

## Conflict of Interest

The authors declare that the research was conducted in the absence of any commercial or financial relationships that could be construed as a potential conflict of interest.
